# Cyclic Loading Performance of Radius-Cut Double Coke-Shaped Strip Dampers

**DOI:** 10.3390/ma13183920

**Published:** 2020-09-04

**Authors:** Jaehoon Bae, Chang-Hwan Lee, Minjae Park, Robel Wondimu Alemayehu, Jaeho Ryu, Youngsik Kim, Young K. Ju

**Affiliations:** 1 School of Civil, Environmental and Architectural Engineering, Korea University, Anamdong, Seongbukgu, Seoul 136713, Korea; skycitybjh80@gmail.com (J.B.); alswo8739@korea.ac.kr (M.P.); robel@korea.ac.kr (R.W.A.); 2 Division of Architectural and Fire Protection Engineering, Pukyong National University, Busan 48513, Korea; chlee@pknu.ac.kr; 3 Technical Research Center, TechSquare Co., Ltd., 25 Banpo-daero, Seocho-gu, Seoul 06710, Korea; jryu@techsq.co.kr (J.R.); yskim@techsq.co.kr (Y.K.)

**Keywords:** reduced beam section, plastic hinge, strip damper, energy dissipation device, cyclic loading, passive control system

## Abstract

Conventional slit dampers are widely used for the purpose of seismic retrofitting, however, the structure of these dampers is susceptible to fractures, due to stress concentration at the ends of the strips in the event of large earthquakes. To address this issue, a novel radius-cut coke-shaped strip damper featuring improved ductility is proposed herein. This damper was developed based on the moment distribution over the strip when both its ends were constrained. The height-to-width ratio of the strip was increased to induce bending rather than shear deformation, and the reduced beam section method was employed. A radius-cut section was used to intentionally focus the stress to induce the plastic hinge. This reduced the fracture fragility of the specimen, resulting in an increased inelastic deformation capacity. Cyclic loading tests were conducted to verify damping performance against earthquakes. Experiments and finite element analyses proved that the coke-shaped damper exhibits improved ductility. The final fracture occurred in the radius-cut section after sufficient energy dissipation during cyclic loading. The results also indicated further improvements in strength due to the membrane effect under cyclic loading, caused by the tensile resistance of the strip due to its constrained ends.

## 1. Introduction

**Figure 1:**
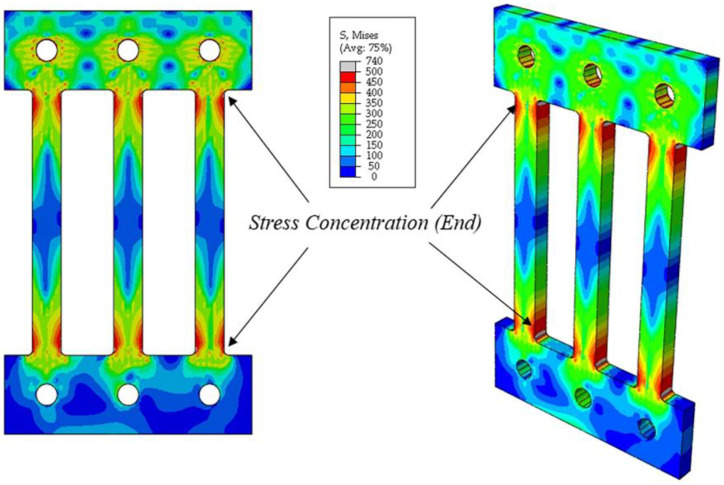
Stress distribution in conventional slip dampers.

Since the Pohang earthquake in 2017, the frequency of earthquakes in the Republic of Korea has increased. Consequently, approaches such as seismic retrofitting and reinforcement have garnered increased research attention. Several studies have also verified the effectiveness of passive damper systems [1,2,3,4,5,6,7,8]. Given that metallic dampers are widely used owing to their cost-effectiveness and ease of replacement [9,10,11,12], several different types of steel dampers have also been proposed. Oh et al. [13] introduced a novel system, wherein a slit damper was employed at the beam-column interface; this damper was easily replaceable and offered improved seismic performance even under the influence of large earthquakes. Kim et al. [14] proposed a cantilever-type steel damper that showed high energy dissipation capability; the ability of this damper to restore stability was experimentally verified. Lee and Kim [15] introduced a box-type steel damper which employed four steel slit plates for seismic retrofitting. However, subsequent studies have indicated that the shape of conventional slit dampers was susceptible to fractures at unintended stages owing to stress concentration at the ends of the strips during large shear deformations [16,17], as illustrated in Figure 1. In addition, recent earthquakes in Japan and California exhibited long-term characteristics; therefore, protection against these effects necessitates dampers with higher ductility [18,19]. To address this need, a novel coke-shaped strip damper is proposed herein. This damper undergoes bending deformation rather than shear deformation, which is achieved by adjusting the width and length ratio of the damper to improve its inelastic deformation capacity. Thus, the proposed damper intentionally concentrates stress at the radius-cut section, facilitating efficient plastic deformation and achieving enhanced ductility. This paper presents the design procedure for the coke-shaped strip damper; the experimental parameters adopted in this study, include the loading of protocol types and the number of plastic hinges for each strip. Moreover, the seismic performance of the proposed damper was verified and compared with results obtained via the finite element method (FEM). The experimental results prove the stable seismic performance of the coke-shaped strip damper in terms of its energy dissipation, equivalent viscous damping, and ductility.

## 2. Radius-Cut Coke-Shaped Strip Damper

**Figure 2:**
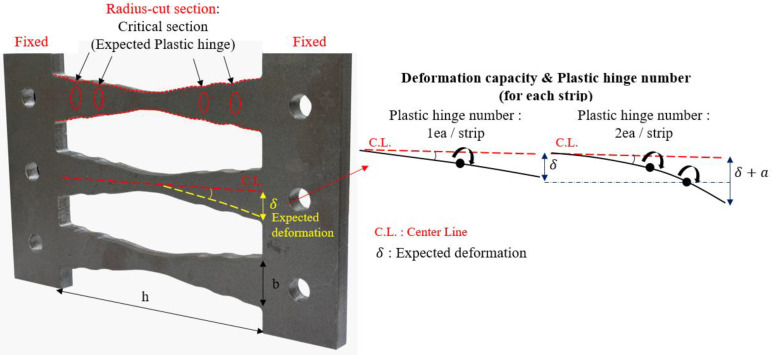
Configuration of the proposed coke-shaped strip damper.

Figure 2 depicts the configuration of the proposed radius-cut coke-shaped strip damper featuring in-plane resistance and comprising fixed end portions and central radius-cut portions. This coke-shaped damper is based on the use of double plates. The height-to-width (*h/b*) ratio of the strip in this damper is set to be greater than five, which corresponds to flexural deformation rather than shear deformation. The fixed portion is connected using frames with high-strength bolts; the shape of the central portion enables a moment distribution that concentrates the stress at the radius-cut section. Therefore, under an efficient plastic deformation, multiple plastic hinges are expected.

This concept of multiple hinges was inspired by research on the reduced beam section (RBS) method, which was conducted after the Northridge Earthquake. The RBS method is used to intentionally generate plastic hinges by reducing the cross-section of the beam in order to increase its ductility and prevent sudden failure [20].

**Figure 3:**
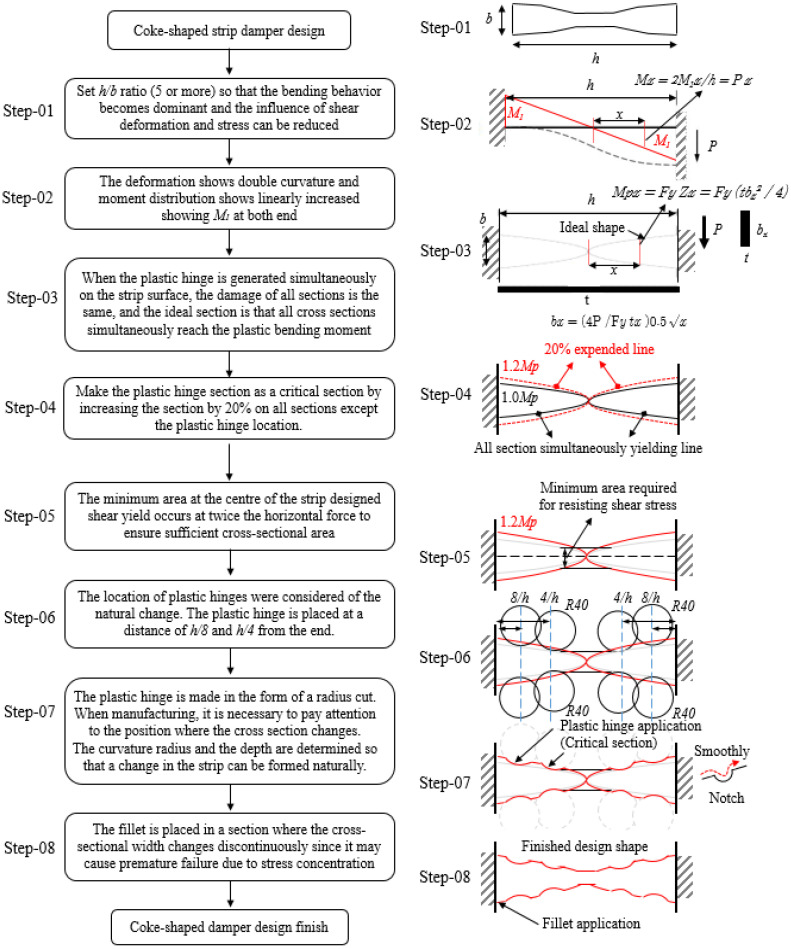
Design procedure for coke-shaped strip damper.

The design procedure for a coke-shaped strip damper is illustrated in Figure 3 and can be detailed as follows:

Step-01. Assuming that the strip damper is subjected to a vertical force (*P*) along the in-plane direction, the damper experiences a combination of bending moment and shear force. However, as the height-to-width ratio of the strip increases, its bending behavior becomes dominant; consequently, the influence of shear deformation and the corresponding stress can be reduced [21,22,23].

Step-02. Assuming that the element restraining the end of the strip is highly resistant, the vertical force is equally distributed over each strip; therefore, each strip can be simplified as an equivalent beam model. Thus, the resultant deformation exhibits double curvature, and the moment distribution is incremented linearly, with moment *M*_1_ at both ends.

Step-03. When a plastic hinge is simultaneously generated on the surface of the strip, the damage over all the sections was found to be identical, which considered as the shape of the damper to the ideal shape. Therefore, the ideal shape of the strip damper requires that all cross-sections are simultaneously subjected to plastic bending moment (*M_p_*).

(1) $$M_{x}=2M_{1}x/h=P\times x$$

(2) $$M_{px}=F_{y}Z_{x}=F_{y}(tb_{x}{}^{2}/4)$$

(3) $$b_{x}={\left(4P/F_{y}t_{x}\right)}^{0.5}\sqrt{x}$$

where *F_y_* is the yield strength of the material; *Z_x_* and *t_x_* are the plastic section modulus and thickness of the strip at a distance of *x* from the center, respectively. *M_x_* is the moment at a distance of *x* from the center, and the corresponding plastic bending moment, *M_px_*, can be calculated using the plastic section modulus (*Z_x_*) at the same position. Moreover, *b_x_* of the strip can be calculated by equating the value of *M_px_* in Equation (2) with that of *M_x_* (=P×x) in Equation (1).

Step-04. For the portion where the plastic hinge was generated, the formula for *M_px_*, suggested in the previous step, was applied; this converted the section with the plastic hinge into a critical section by increasing the area of all regions by 20%, except at the location of the plastic hinge.

Step-05. The design of the strip damper, with *h/b* ≥ 5, was primarily based on resistance to the required moment without considering shear force. Even if the shear force has a negligible effect at the center of the strip, the membrane effect occurs because both ends of the strip are firmly fixed. In particular, for a large deformation, additional vertical stresses are generated due to the tensile resistance effect (i.e., the membrane effect). Therefore, the shape needs to be adjusted such that the central portion of the strip has sufficient cross-sectional area to prevent the occurrence of plastic hinges and brittle fractures at the center. Therefore, the minimum required area at the center of the strip was determined considering that the width of the strip influences the condition that the shear yield is twice the horizontal force; this ensures a sufficient cross-sectional area at the center of the strip.

Step-06. The number of plastic hinges on each side was considered to be one and two, according to the natural change and processability of the section. The discussion here in considers two plastic hinges on each side of the strip; these plastic hinges are placed at a distance of h/8 and h/4 from the ends of the strip. The cross-section of these plastic hinges is designed as a circular arc with a constant radius of curvature of R40 mm.

Step-07. The plastic hinges are designed using a radius cut, which forms the critical section. Precise manufacturing of the position where the cross-section changes is necessary to ensure that the notch remains as undamaged as possible. The radius-cut design assumes an area of *b* × *h*. The width and depth of the notch are determined based on the location where stress concentration occurs, which was determined via FEM simulations using Abaqus (Dassault systems, Waltham, Boston, MA, USA). The curvature and depth of the notch are determined such that the desired strip width can be formed naturally.

Step-08. In the area where the radius of the plastic hinge is shaped in the form of a radius cut, a fillet is placed in the section where the cross-sectional width discontinuously varies because it may cause premature failure, which is caused by stress concentration and deterioration in the area where the strip width varies significantly.

## 3. Experimental Procedure

### 3.1. Test Specimens

**Figure 4:**
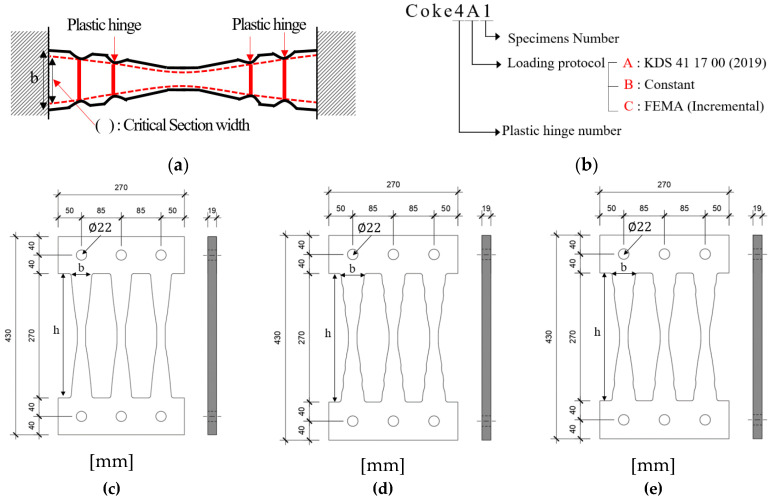
Test specimens. (**a**) Critical section width; (**b**) Specimens name; (**c**) 1.0 *M_p_* acting on all sections simultaneously; (**d**) COKE4 (4 plastic hinges per strip); (**e**) COKE2 (2 plastic hinges per strip).

Different types of cyclic loading tests were conducted to investigate the structural performance and fatigue characteristics of the coke-shaped strip damper. A total of 13 damper specimens with different shapes and damping forces were used in this study. These specimens were categorized as COKE4 and COKE2, according to the number of plastic hinges on the strips of the dampers; a value of 1.0 *M_p_* was selected for comparison with other coke-shaped dampers through an ideal experiment, wherein the plastic moment was simultaneously realized in all sections. Table 1 and Figure 4 present specifications of the test specimens.

### 3.2. Loading Protocol

**Figure 5:**
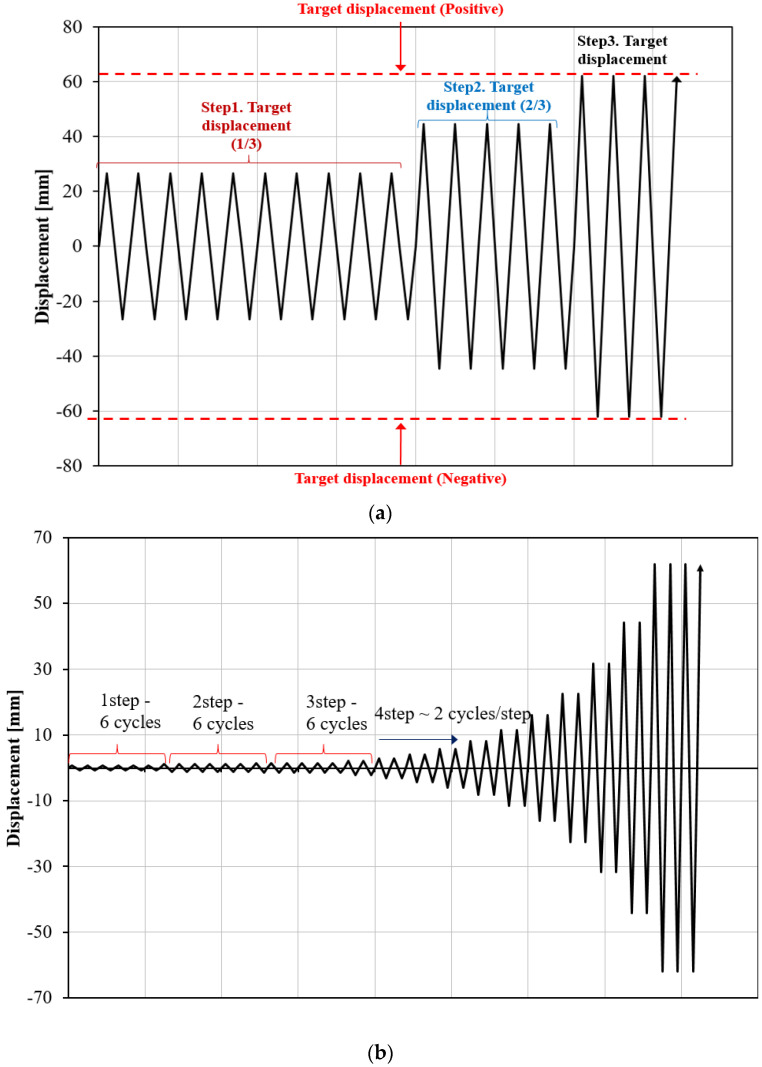

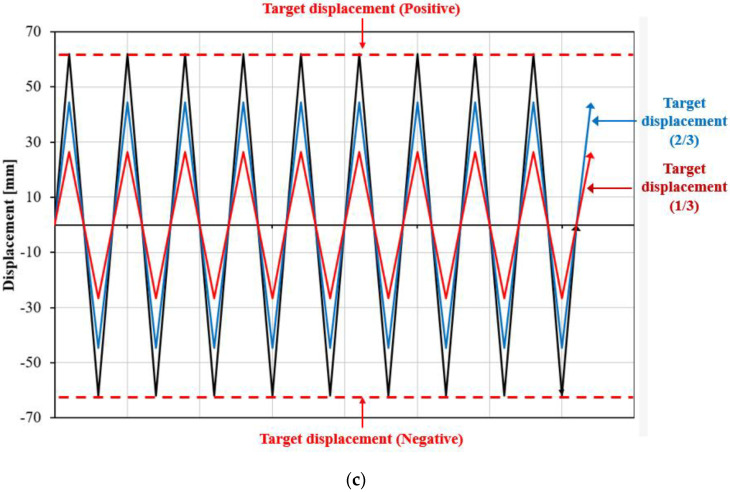
Loading protocols used in the study. (**a**) KDS 41 17 00 (2019); (**b**) FEMA (incremental loading); (**c**) Constant loading.

Force was applied on the specimens using the displacement control method; Figure 5 depicts the protocol used in this paper, including KDS 41 17 00 [24] which is the same as the AISC loading protocols [25,26], an incremental FEMA (Federal Emergency Management Agency) [26], and a constant loading used during the tests. The purpose of this loading was to verify the performance of the damper under different loading conditions. A constant load was also applied to investigate damper fatigue. Under the KDS 41 17 00 (2019) protocol, for displacement-dependent steel dampers, the maximum displacement of the target building was confirmed after analyses based on the maximum considered earthquake (MCE) level. When a displacement corresponding to one-third of the target displacement was applied 10 times, a displacement corresponding to two-thirds of the target displacement was applied 5 times, and a displacement corresponding to the target displacement was applied more than 3 times, the performance of the target was considered to be satisfactory if the damper load did not decrease by more than 15% of the maximum load; a reduction exceeding 15% of the maximum load was considered to be damper failure.

For the experiment, the relative displacements of the actuator and the damper needed to be calibrated, considering the sliding of a bolted joint. The target displacement was corrected through three trials of the actual test. On the basis of the results, it was confirmed that the specimen could sufficiently achieve the target displacement of 50 mm, provided the forced displacement was set as 62 mm.

Under the loading protocol of FEMA 461 [27], the target displacement ∆*m* was set as 62 mm, based on the test results, and the amplitude was increased to 1.4 times the amplitude of the previous step. In addition, the amplitude of each forced step was designed such that the target displacement could be achieved at the 14th step. From the first to the third steps, six cycles of amplitudes were considered under an elastic state; thereafter, two cycles were repeated for each step. Constant loading was also considered to determine the fatigue of the coke-shaped strip dampers.

### 3.3. Experimental Setup

**Figure 6:**
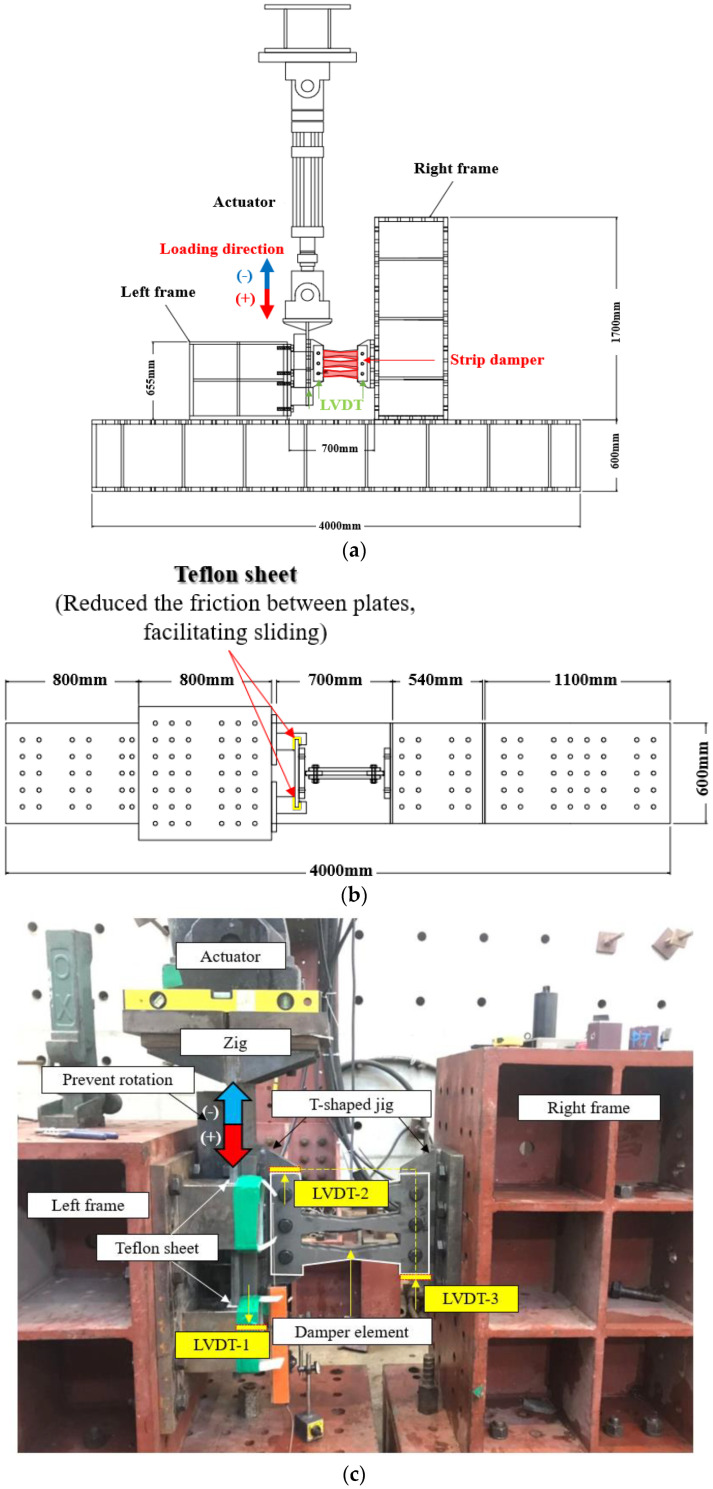
Experimental setup. (**a**) Front side; (**b**) Top; (**c**) Test setup.

Figure 6 presents the test setup. Considering that the force generated at the connections increased due to the tensile resistance of the strip (i.e., the membrane effect), sufficient bolts were employed in the setup. The experiments were performed using a 1000 kN actuator. The damper was fixed to the left and right frames using a T-shaped Zig, and a high-strength bolt was used to affix the two zigs. To prevent vertical displacement of the T-shaped jig, a Teflon sheet was placed in the gap between the zig and the left and right plates; this sheet also reduced the friction between plates, facilitating sliding. A backplate was also utilized to prevent rotation and to transfer the load from the actuator to the test specimen. A damper plate was installed behind the zig to minimize the generation of moment, thereby ensuring that the applied load was transferred from the actuator to the test specimen.

### 3.4. Material Properties

The tensile specimens were prepared according to No. 1A (KS B 0801), and the tensile tests were conducted in accordance with the test standards of KS B 0802 [28]. SS275 steel was used in this experiment, and the test results indicated that the specimens exceeded the lower yield strength limit of 265 MPa, as specified in KS D 3503 [29]; the tensile strength of this steel was also within permissible limits.

## 4. Experimental Results

**Figure 7:**
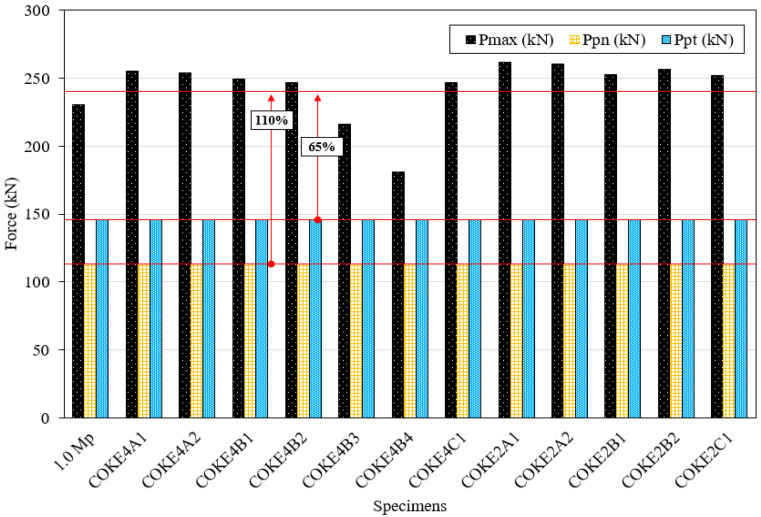
Comparison of test results and theoretical values.

Table 2 and Figure 7 present a comparison between the theoretical and experimental results. The average value of *P_max_* was 240 kN, which was 110% greater than the value of *P_pn_* and 65% greater than the value of *P_pt_*. The results indicate that the strength of the coke-shaped strip damper exceeds the theoretical plastic strength. The experimental value was considerably higher than the theoretical value; moreover, the applied load was greater than the expected load due to the effect of tensile resistance, caused by the two fully constrained ends.

### 4.1. Load-Displacement Relationships

**Figure 8:**
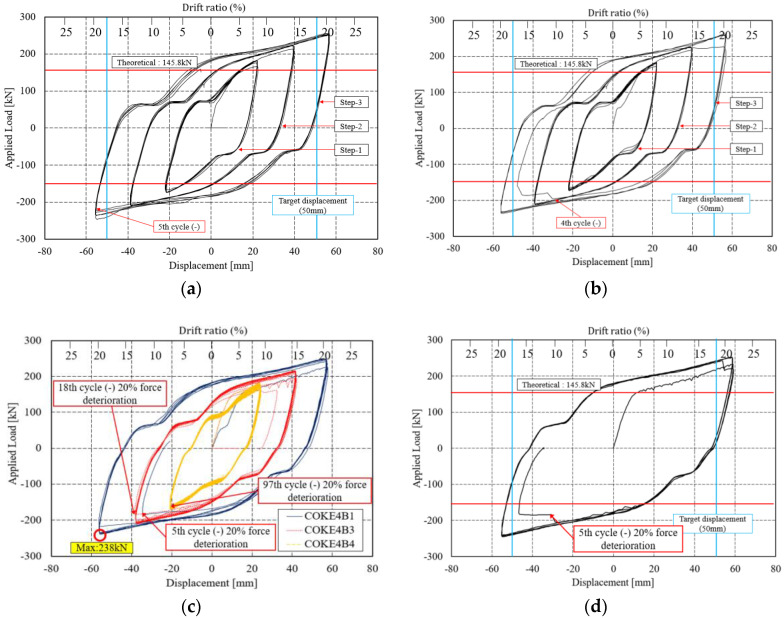

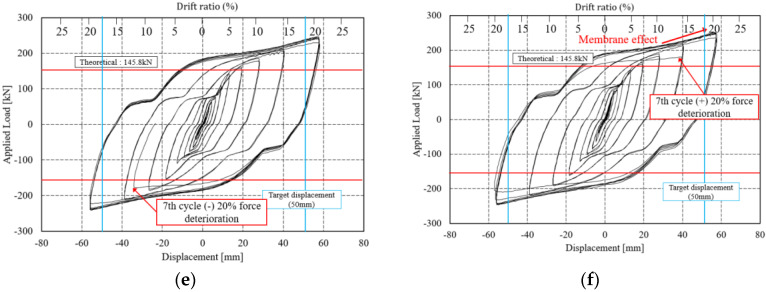
Load-displacement relationships for COKE2 and COKE4. The red horizontal lines indicate the theoretical force, whereas the blue vertical lines indicate the target displacement of the damper. (**a**) COKE4A1; (**b**) COKE2A1; (**c**) COKE4B1-4; (**d**) COKE2B1; (**e**) COKE4C1; (**f**) COKE2C1.

The load-displacement curves of the specimen and the damper drift ratio are plotted in Figure 8. In all the experiments, the slip occurring during the first load is negligible because the bolt is positioned downward and exerts a downward force. During the first loading cycle, the bolt remained fixed; subsequently, it started to deform, which consequently resulted in noise and eventually led to a bolt slip. Figure 8a,b illustrates the applied load and displacement curves when the loading is in accordance with the KDS 41 17 00 (2019) standard. The force reduction in both specimens (i.e., COKE 4 and COKE2) occurred during the third step. For COKE4, it occurred during the fifth cycle in the negative direction, whereas for COKE2, it occurred during the fourth cycle in the negative direction. The experiment was terminated after a force reduction of 20% of the maximum force. According to the regulations of AISC 7–16 and KDS 41 17 00, the target displacement of the damper device is based on the expected device displacement during the maximum considered earthquake. The damper performance is considered to be satisfactory if the damper does not break for ten times at 0.33 times the target displacement, five times at 0.67 times the target displacement, and three times at 1.0 times the target displacement. In accordance with these criteria, the proposed coke-type strip damper was discontinued after the performance requirements were satisfied.

Cumulative inelastic deformation can deteriorate damper performance; thus, the performance of the damper was verified using a low-cycle fatigue test. Figure 8c,d present the graph for the low-cycle fatigue, indicating that that the in-plane coke-shaped damper exhibits stable behavior under a low-frequency cyclic loading. In particular, as shown in Figure 8c, the stable behavior of the damper was verified based on the resistance of the damper toward accumulated fatigue through repeated tests, for displacements corresponding to one-third and two-thirds of the maximum target displacement, as well as the total target displacement [30].

COKE4B1 and COKE2B1 were damaged after five cycles under the maximum displacement. However, the maximum displacement of a given target is calculated considering the MCE level; this is set considering a return period of 2475 years (i.e., 2% in 50 years), which rarely occurs in actual scenarios. Moreover, for a target displacement of 50 mm, the maximum strain of the damper exceeds 18% drift ratio. In this case, the damper is sufficiently deformed. Moreover, if the damper is damaged after five cycles, the damper is said to be sufficiently deformed, although in the KBC2016 testing standard, damper performance is deemed satisfactory if the load does not fall below 15% of the maximum load, over five cycles under the maximum displacement [31]. In addition, the structural safety of the damper against progressively increasing loads was tested using the incremental force loading protocol proposed by FEMA. Figure 8e,f presents the results for the incremental force experiments; these results yield a stable hysteresis curve. For the COKE4C1 test specimen, a force reduction of approximately 5% occurred during the sixth negative cycle; during the seventh positive cycle, the force was reduced significantly by an additional 20%. The experiment was ceased at this point. The behavior of the COKE2C1 test specimen was similar to that of the COKE4C1 specimen; however, the force reduction was initialized earlier than that in the COKE4 specimen, and a reduction of approximately 5–10% occurred during the sixth negative cycle. Moreover, the force was reduced significantly by more than 20% during the seventh positive cycle, and the experiment was terminated. For all specimens, the load increased beyond the theoretical value due to the fully constrained ends of the specimens.

### 4.2. Initial Stiffness and Yield Strength

**Figure 9:**
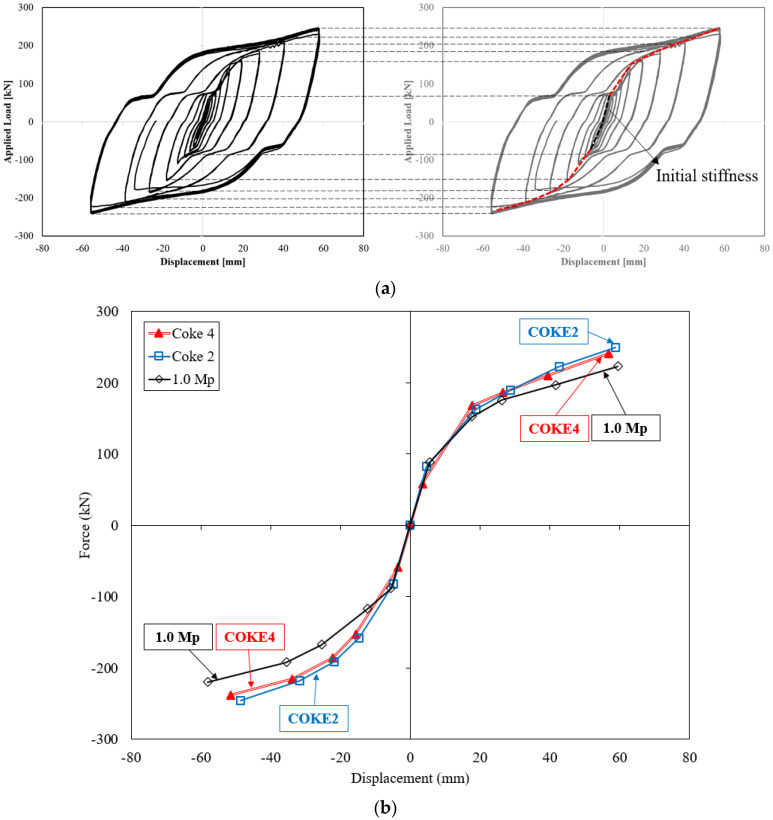
Skeleton curves. (**a**) Concept of skeleton curve; (**b**) Skeleton curve of specimens.

Figure 9 presents the skeleton curve that was used for an accurate comparison between the initial stiffness and yield strength. This monotonic curve was produced by connecting the maximum force in each cycle of the hysteresis loop; the results coincide with the monotonic curve for the static load test. This was used as an index to estimate the plastic deformation capacity by Kato [32]. The stiffness, which is one-third of the initial stiffness, is translated to a vertical axis and the point in contact with an arbitrary line of the load-displacement curve is evaluated as the yield point by Moehel’s method. The skeleton curves of the COKE4 and COKE2 specimens are highly similar; the maximum force of COKE4 lies between those of the COKE2 specimen and the 1.0 *M_p_* model. According to the skeleton curve, the yield displacement and stiffness of the damper were found to be 5.5 mm (yield force 88 kN) and 16.0 kN/mm for the 1.0 *M_p_* specimen, 4.73 mm (yielding force 82.4 kN) and 17.4 kN/mm for the COKE2 specimen, and 3.47 mm (yielding force 58.3 kN) and 16.8 kN/mm for the COKE4 specimen, respectively. The yield point of the coke-shaped damper exhibited a tendency to occur earlier as the number of cross-sectional defects (i.e., plastic hinges) increased.

### 4.3. Effective Stiffness and Equivalent Damping

**Figure 10:**
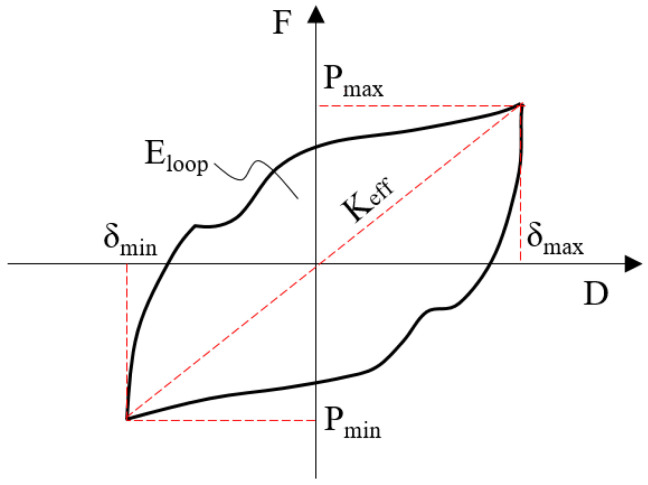
Effective stiffness and energy dissipation during a cycle.

Equivalent damping can be used to estimate the nonlinear damping ability for a non-velocity damper such as a metallic damper. Figure 10 depicts the effective stiffness and energy dissipation of a coke-shaped strip damper during a cycle [16,33].

The equivalent stiffness and viscous damping can be calculated using Equations (4) and (5), respectively:

(4) $$K_{eff}=\frac{\left|P_{\max}\right|+\left|P_{\min}\right|}{\left|\delta_{\max}\right|+\left|\delta_{\min}\right|}$$

where *δ*_max_ and *δ*_min_ are the positive and negative peak displacements, and *P*_max_ and *P*_min_ are the positive and negative forces at *δ*_max_ and *δ*_min_, respectively.

(5) $$\beta_{eff}=\frac{2}{\pi }\frac{E_{loop}}{K_{eff}{\left(\left|\delta^{+}\right|+\left|\delta^{-}\right|\right)}^{2}}$$

where $\beta_{eff}$ is the equivalent damping ratio, $K_{eff}$ is the effective stiffness of the damper, and $E_{loop}$ is the energy dissipation area in the loop in Figure 10. 

**Figure 11:**
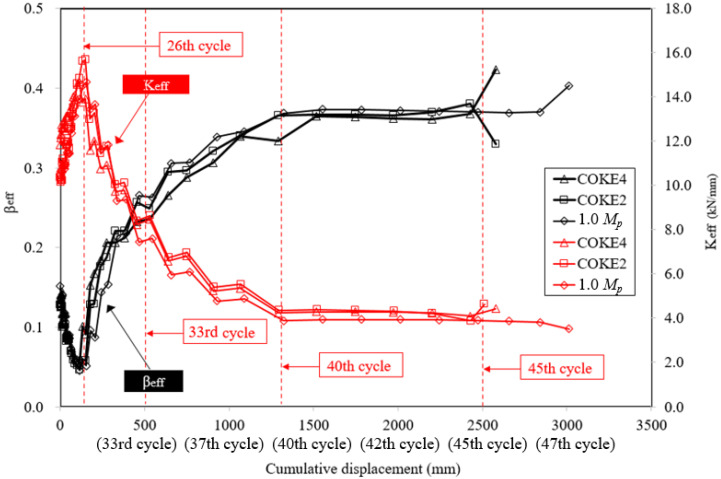
Effective stiffness and effective damping for the experiments.

**Figure 12:**
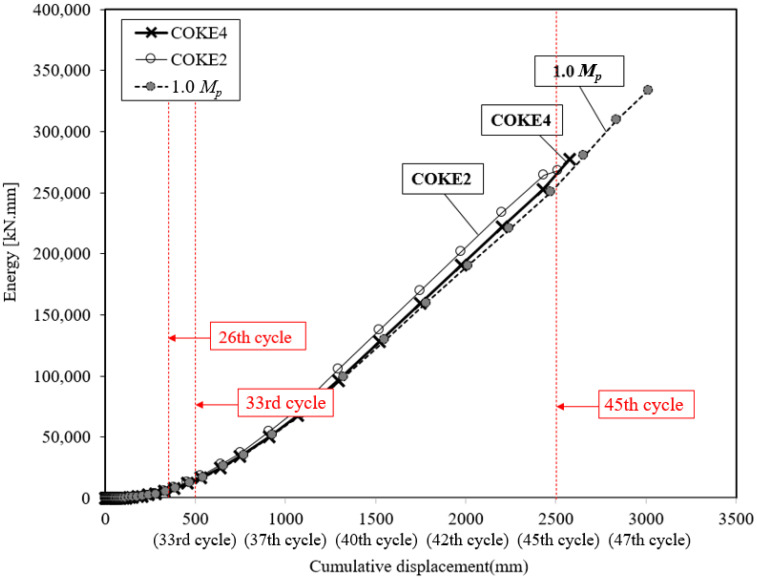
Energy dissipation capacity comparison.

**Figure 13:**
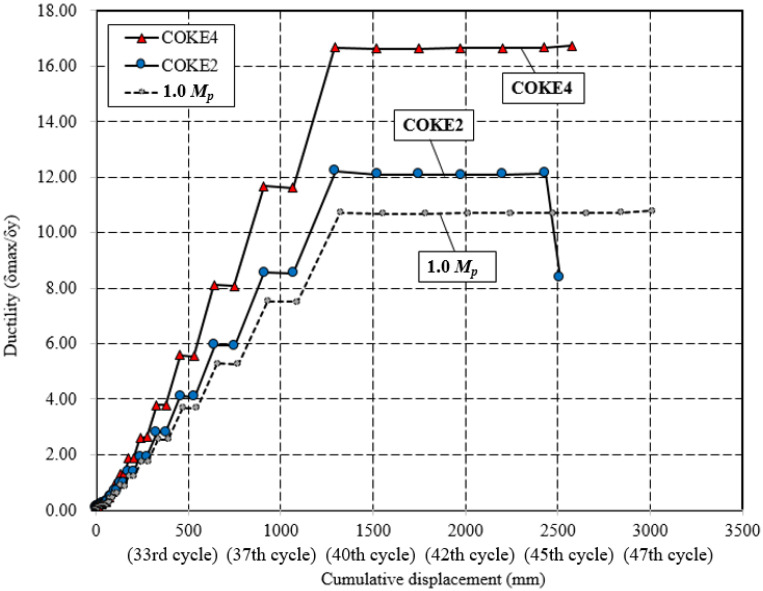
Comparison of ductility under cyclic loading.

Figure 11 presents the effective stiffness ($K_{eff}$) and effective damping ($\beta_{eff}$). The effective stiffness gradually decreases as the cumulative plastic deformation increases; the maximum effective stiffness appears during the 26th cycle, and the effective damping range is between 5% and 40%, which are opposite trends with the effective stiffness. During the 26th cycle, the lowest effective damping was observed; furthermore, as the cumulative number of cycles increased, after the 26th cycle, the effective stiffness of the damper gradually decreased, and the damping and the slope of the energy dissipation capacity increased (Figure 12). The stiffness gradually decreases, the damping gradually increases, and the graph crosses at the 33rd cycle. While experiencing cumulative deformation corresponding to the 40–45th cycles, there was a negligible decrease in damping, and it did not increase significantly until the occurrence of fractures after the 45th cycle. Moreover, in this region, ductility remained constant, although the damper was destroyed (Figure 13).

### 4.4. Energy Dissipation Capacity

Figure 12 presents the energy dissipation under the incremental loading protocol. The results indicated that both the COKE4 and COKE2 specimens exhibited considerably similar results and that the energy dissipation capacity of the coke-shaped damper was higher than that of the 1.0 *M_p_* damper for all the cycles. However, the 1.0 *M_p_* damper sustained a larger number of loading cycles than that of the coke-shaped damper; therefore, the cumulative dissipation energy of the 1.0 *M_p_* damper was higher.

**Figure 14:**
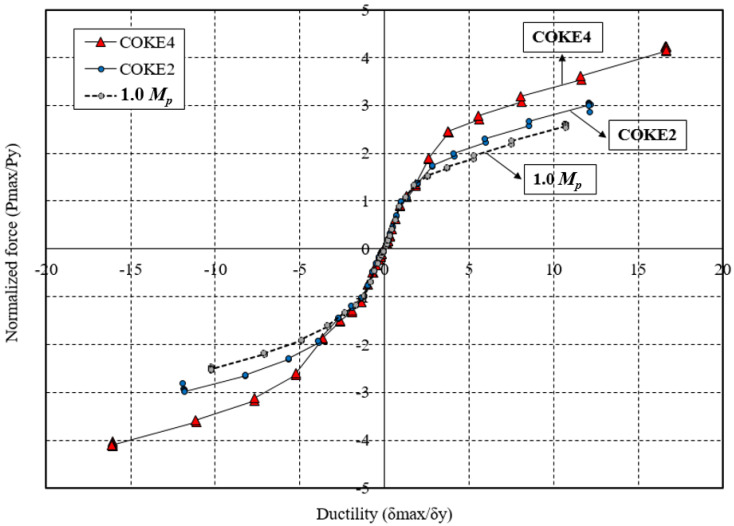
Ductility and normalized force.

Figure 13 presents the variation in the ductility of each test specimen with respect to the cumulative strain. Specifically, it compares the ductilities of the 1.0 *M_p_*, COKE4, and COKE2 models for all cycles. COKE4 exhibits a more gradual change in ductility as compared with the 1.0 *M_p_* and COKE2 models. When a plastic hinge was created due to an intentional defect in the cross-section, the initial yield point was achieved faster. However, when conditions of the cross-section were identical, it was observed that the final fracture point occurred in a similar manner. Furthermore, in terms of ductility, the COKE4 model with four plastic hinges per strip was superior to the COKE2 model with two plastic hinges per strip. Thus, it was confirmed that an increase in the number of plastic hinges led to improved ductility. However, the number of specimens was considerably limited; therefore, additional FEM analyses were conducted, as detailed in Section 4.5, to estimate the effect of the number of plastic hinges on ductility. Figure 14 indicates the difference in the load resistance capability of each specimen for the same ductility. In this case, the normalized force (*P_max_*/*P_y_*) was the highest for COKE4, and this was followed by COKE2. The 1.0 *M_p_* test specimen exhibited the lowest load resistance because its cross-sectional area was 20% smaller than those of the other specimens.

### 4.5. FEM Analysis and Effect of Number of Plastic Hinges

On the basis of the results of this experiment, the FEM analyses for three types of damper models were conducted using the Abaqus CAE commercial software. To simulate the membrane effect caused by the fixed ends, virtual springs were installed at both ends of the strip damper. The finite elements were modeled using yield stress, ultimate stress, and Young’s modulus values of 341 MPa, 515 MPa, and 176 GPa, respectively. The values of these mechanical properties were obtained via a coupon test on the material of the strip damper. The stress–strain relation obtained from the coupon test was converted to the true stress and true strain and assigned to the finite element models. The finite element models were meshed using an eight-node linear brick element with reduced integration. The average mesh size was 4 mm.

**Figure 15:**
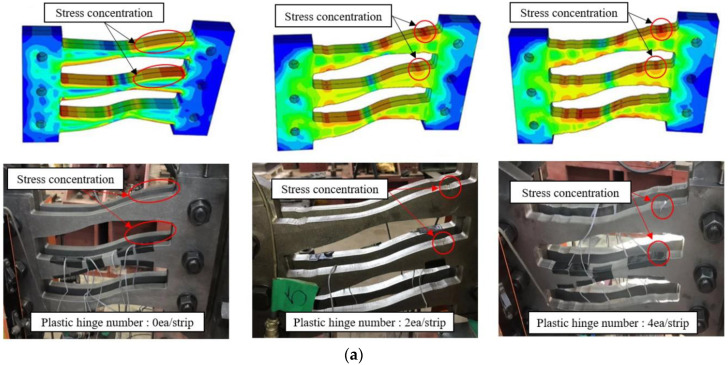

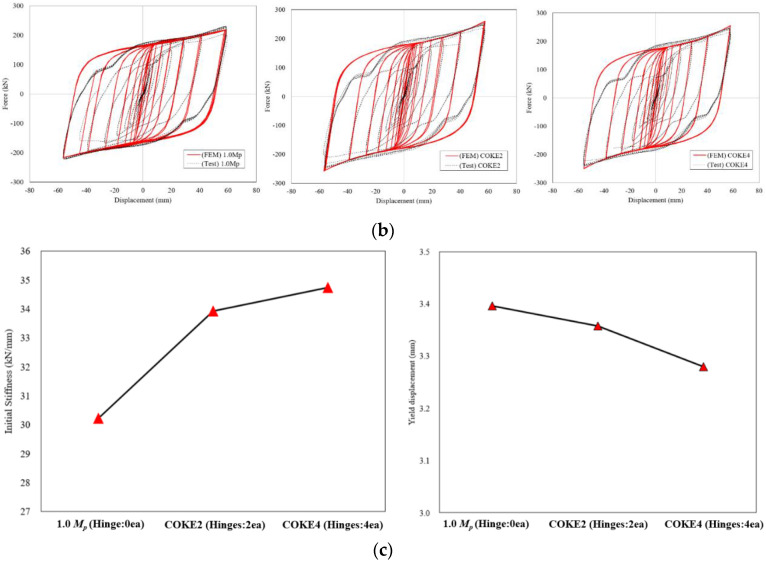
Results of the FEM analyses. (**a**) von Mises stress distribution in the FEM and experimental results; (**b**) Force and displacement graphs of the FEM and experimental results; (**c**) Initial stiffness and yield displacements obtained from FEM results.

Figure 15a depicts the von Mises stress distribution for the three analysis models, and Figure 15b presents comparisons between the applied load and displacement obtained via analyses and the experimental results. It was confirmed that the plastic hinge occurs at the radius-cut segment of the coke-shaped strip damper, according to the design of the plastic hinge. Moreover, the load-displacement curves obtained using experimental and analytical values exhibited stable behavior. However, the FEM analysis and the load-displacement curve of the actual experiment exhibited minor differences during the reloading process. This was because, in the FEM analysis, the slip of the bolt under unloading and reloading cycles was not considered. This bolt slip, however, occurred during the actual test. Figure 15c illustrates the initial stiffness and yield displacements for the following three specimens: 1.0 *M_p_* (plastic hinge 0 ea), COKE2 (plastic hinge 2 ea/strip), and COKE4 (plastic hinge 4 ea/strip). According to the FEM results, the initial stiffness of the coke-shaped damper was greater than that of the 1.0 *M_p_* model, because the increased stiffness and maximum force increased the strip width by 20%. Furthermore, a comparison of the coke-shaped slit dampers showed that the initial yield was achieved faster as the number of plastic hinges increased; this was likely caused by the decrease in cross-section. These characteristics are in-line with the experimental results. The energy dissipation capacity of the coke-shaped strip damper was also higher than that of the 1.0 *M_p_* model, for all displacement values. Moreover, the coke-shaped model consumed more energy owing to its increased resistance. However, the 1.0 *M_p_* model sustained a larger number of loading cycles as compared with the coke-shaped strip dampers; therefore, the cumulative dissipation energy of this strip damper was higher.

### 4.6. Failure Modes

**Figure 16:**
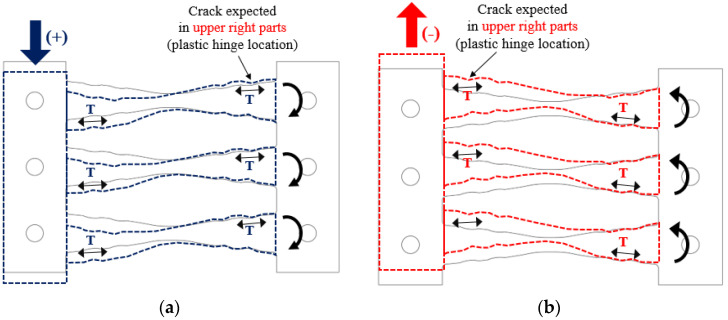
Damper behaviors. (**a**) under positive applied force; (**b**) under negative applied force.

Figure 16 illustrates the deformation and fracture of the specimens. The fracture of the specimens initializes with noise, and small cracks are formed within the vicinity of the plastic hinges as the amplitudes of the specimens increase. The upper-right portion of each strip is subjected to tension under a positive force (downward), whereas the upper-left portion is subjected to tension under a negative force (upward). The cracks gradually deepen and result in the formation of a fracture. This leads to a rapid reduction in strength, after which the experiment was terminated. It was confirmed that cracks were generated at the plastic hinges on the upper-right portion of the strip during applied positive and negative forces. In addition, cracks were created at the upper-left portion, which was subjected to negative tensile force. The fracture phenomena in all specimens were similar.

**Figure 17:**
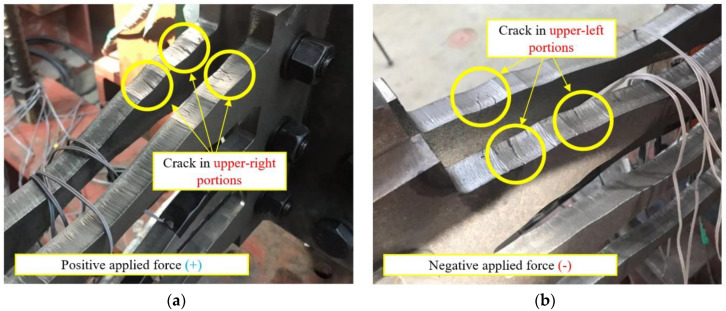
Cyclic behavior and failure of the COKE damper. (**a**) under positive applied force; (**b**) under negative applied force.

Figure 17 shows the cyclic behavior and failure of the COKE4 and COKE2 dampers, respectively. It can be seen that cracks occur at the plastic hinges on the upper-right portion of the strip, under the application of a positive force (Figure 17a, COKE4, Step 14, seven cycles), or the upper-left portion, under the application of a negative force (Figure 17b, COKE2, Step 14, six cycles).

**Figure 18:**
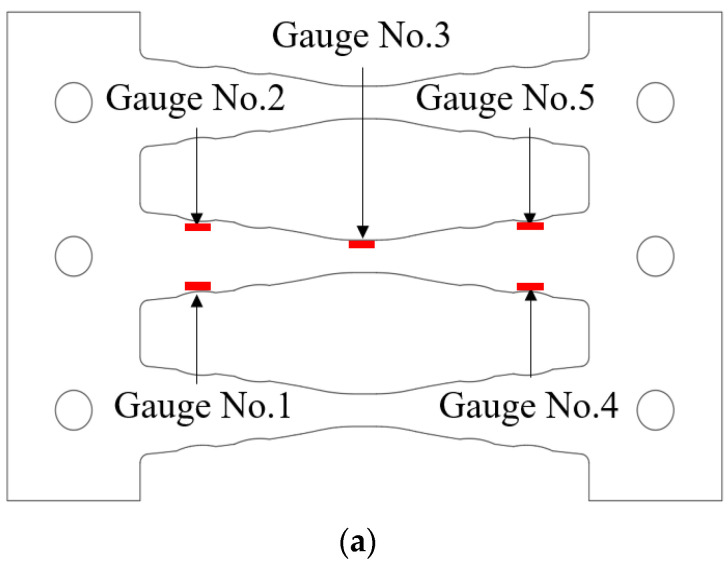

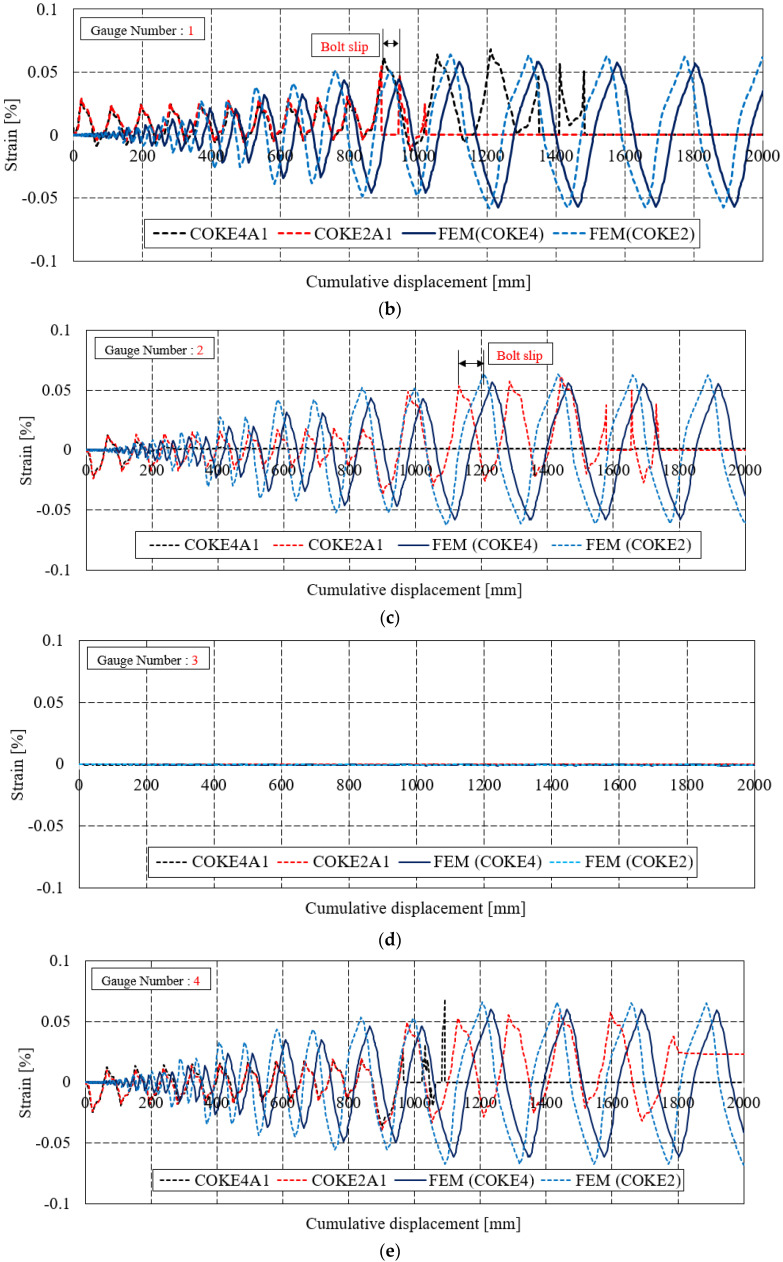

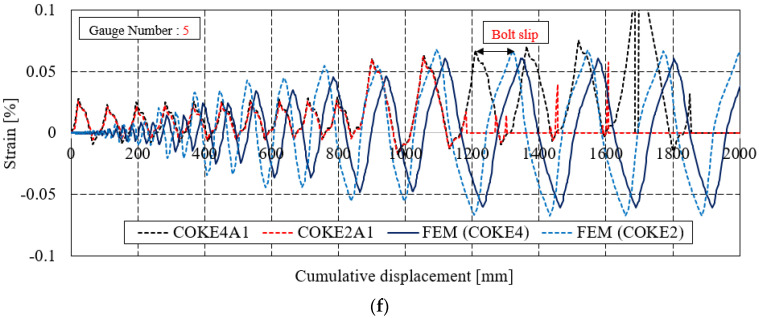
Strain distribution and cumulative displacements. (**a**) Gauge number position; (**b**) Gauge No. 1; (**c**) Gauge No. 2; (**d**) Gauge No. 3; (**e**) Gauge No. 4; (**f**) Gauge No. 5.

Figure 18 depicts the strain distribution on a strip of the damper with respect to the cumulative displacements. The results of both COKE4 and COKE2, under the KDS 41 17 00 (2019) loading protocol, exhibit similar trends. Neglecting the deviation due to bolt slip, the FEM analysis results were almost identical to the experimental values, at the maximum strain. Gauge No. 3 represents the specimen behavior within the elastic range, and Gauge Nos. 1, 2, 4, and 5 denote those within the plastic range. In addition, the results show that the strip damper exhibits flexural behavior at both ends, and a plastic hinge was generated in the reduced cross-section. FEM analysis was used to confirm stress concentration; the definition of specimen fracture was not considered in the analysis. It was confirmed through the FEM stress distribution that it exceeded the yield in the radius-cut region and was in the plastic region. Fractures occurred and indicated the early termination of gauge response as compared with the FEM response for some of the gauges.

## 5. Conclusions

Conventional slit dampers can undergo fractures owing to stress concentration at the ends of the strip, when subjected to large seismic motions. To overcome this issue, this paper proposed a novel radius-cut coke-shaped strip damper featuring increased ductility. The coke-shaped strip damper has an optimized shape based on the moment distribution over the strip when both its ends are fully constrained. Moreover, the height-to-width ratio of the strip was increased to induce bending deformation rather than shear deformation; additionally, the RBS method was implemented for the radius-cut section to ensure that fractures affected all sections equally. This increased the inelastic deformation by reducing the fracture fragility of the specimen. Moreover, cyclic loading tests under different loading protocols were conducted to verify the seismic performance of the model against earthquakes. The results thus obtained are described below:

1. The critical section of the coke-shaped strip damper was the radius-cut section where the plastic hinge was located. The observed maximum strength was 65% greater than its theoretical value when using nominal strength and 110% greater than the theoretical value when using the test yield strength. It was confirmed that the membrane effect enhanced the maximum strength when both ends were fixed.
2. The target displacement was calibrated by considering actual bolt slip at both ends through trials, and a variety of loading protocols were used to verify damper performance. Moreover, it was confirmed that the coke-shaped strip damper satisfied the performance criteria and exhibited stable behavior, as evidenced by the displacement-force curve.
3. During the design of the coke-shaped damper, the total cross-section was increased by 20%. Therefore, the maximum force of COKE2 and COKE4 was greater than that of 1.0 *M_p_*. Moreover, the COKE4 model, which featured an increased number of plastic hinges, yielded faster than the COKE2 and 1.0 *M_p_* models. Thus, it was concluded that ductility increased with the number of plastic hinges.
4. COKE2 and COKE4, featuring increased cross-sections, exhibited similar initial stiffnesses, whereas that of the 1.0 *M_p_* model was relatively low. The energy dissipation capacity for the COKE4, COKE2, and 1.0 *M_p_* models increased gradually. The results indicated that both the COKE4 and COKE2 specimens exhibited considerably similar results and that the energy dissipation capacity of the coke-shaped damper was higher than that of the 1.0 *M_p_* damper for all the cycles. However, the highest cumulative energy dissipation was observed for the 1.0 *M_p_* model after 45 cycles. A comparison of ductility and the normalized force confirmed that the maximum load increased as the number of hinges increased, for the same value of ductility.
5. In this study, the number of plastic hinges was limited to two and four per section, owing to limitations on the length and construction of the sections. Thus, further studies should consider the effect of multiple plastic hinges on the damping capacity, energy dissipation capacity, and stiffness and ductility of the damper.

## Figures and Tables

**Table 1:** Specifications of the specimens.

Specimens	Material	Thickness (mm)	*b* (mm)	*h* (mm)	No. of Steel Plates (EA)	Plastic Hinge (EA)	Loading Protocol
1.0 *M_p_*	SS275	19	45	270	2	0	FEMA (Incremental)
COKE4A1	SS275	19	49 (45)	270	2	4	KDS 41 17 00 (2019)
COKE4A2	SS275	19	49 (45)	270	2	4	KDS 41 17 00 (2019)
COKE4B1	SS275	19	49 (45)	270	2	4	Constant (62 mm)
COKE4B2	SS275	19	49 (45)	270	2	4	Constant (62 mm)
COKE4B3	SS275	19	49 (45)	270	2	4	Constant (44.5 mm)
COKE4B4	SS275	19	49 (45)	270	2	4	Constant (26.5 mm)
COKE4C1	SS275	19	49 (45)	270	2	4	FEMA (Incremental)
COKE2A1	SS275	19	49 (45)	270	2	2	KDS 41 17 00 (2019)
COKE2A2	SS275	19	49 (45)	270	2	2	KDS 41 17 00 (2019)
COKE2B1	SS275	19	49 (45)	270	2	2	Constant (62 mm)
COKE2B2	SS275	19	49 (45)	270	2	2	Constant (62 mm)
COKE2C1	SS275	19	49 (45)	270	2	2	FEMA (Incremental)

Note, the values in parentheses denote the critical widths of the section.

**Table 2:** Comparison between test results and theoretical values.

Specimen	*F_yn_* (MPa)	*F_yt_* (MPa)	*P_max_* (kN)	*P_pn_* (kN)	*P_pt_* (kN)	*P_max_*/*P_pn_*	*P_max_*/*P_pt_*
1.0 *M_p_*	265.0	341.0	230.2	113.3	145.8	2.03	1.57
COKE4A1	265.0	341.0	255.3	113.3	145.8	2.25	1.75
COKE4A2	265.0	341.0	253.9	113.3	145.8	2.24	1.74
COKE4B1	265.0	341.0	249.5	113.3	145.8	2.20	1.71
COKE4B2	265.0	341.0	247.1	113.3	145.8	2.18	1.69
COKE4B3	265.0	341.0	216.2	113.3	145.8	1.91	1.48
COKE4B4	265.0	341.0	181.3	113.3	145.8	1.60	1.24
COKE4C1	265.0	341.0	246.8	113.3	145.8	2.18	1.69
COKE2A1	265.0	341.0	262.0	113.3	145.8	2.31	1.80
COKE2A2	265.0	341.0	260.2	113.3	145.8	2.30	1.78
COKE2B1	265.0	341.0	252.7	113.3	145.8	2.23	1.73
COKE2B2	265.0	341.0	256.5	113.3	145.8	2.26	1.76
COKE2C1	265.0	341.0	251.7	113.3	145.8	2.22	1.73

*F_yn_*, nominal yield strength; *F_yt_*, test yield strength of the material; *P_max_*, experimental results at maximum strength; *P_pn_*, theoretical plastic strength calculated using nominal yield strength; *P_pt_*, theoretical plastic strength calculated using the test yield strength.
